# G9a/GLP Complex Maintains Imprinted DNA Methylation in Embryonic Stem Cells

**DOI:** 10.1016/j.celrep.2016.03.007

**Published:** 2016-03-24

**Authors:** Tuo Zhang, Ausma Termanis, Burak Özkan, Xun X. Bao, Jayne Culley, Flavia de Lima Alves, Juri Rappsilber, Bernard Ramsahoye, Irina Stancheva

**Affiliations:** 1 Wellcome Trust Centre for Cell Biology, University of Edinburgh, Michael Swann Building, Max Born Crescent, Edinburgh EH9 3BF, UK; 2 Institute of Genetics and Molecular Medicine, The University of Edinburgh, Western General Hospital, Crewe Road South, Edinburgh EH4 2XR, UK; 3 Department of Bioanalytics, Institute of Biotechnology, Technische Universität Berlin, 10623 Berlin, Germany

## Abstract

DNA methylation at imprinting control regions (ICRs) is established in gametes in a sex-specific manner and has to be stably maintained during development and in somatic cells to ensure the correct monoallelic expression of imprinted genes. In addition to DNA methylation, the ICRs are marked by allele-specific histone modifications. Whether these marks are essential for maintenance of genomic imprinting is largely unclear. Here, we show that the histone H3 lysine 9 methylases G9a and GLP are required for stable maintenance of imprinted DNA methylation in embryonic stem cells; however, their catalytic activity and the G9a/GLP-dependent H3K9me2 mark are completely dispensable for imprinting maintenance despite the genome-wide loss of non-imprinted DNA methylation in H3K9me2-depleted cells. We provide additional evidence that the G9a/GLP complex protects imprinted DNA methylation by recruitment of de novo DNA methyltransferases, which antagonize TET dioxygenass-dependent erosion of DNA methylation at ICRs.

## Highlights

• ESCs lacking G9a and GLP display loss of DNA methylation from ICRs
• The enzymatic activity of G9a/GLP is dispensable for imprinted DNA methylation
• G9a/GLP stabilize imprinting by recruitment of de novo DNA methyltransferases to ICRs
• Recruitment of DNMTs to ICRs antagonizes TET-dependent loss of DNA methylation

## Graphical Abstract

**Figure:**
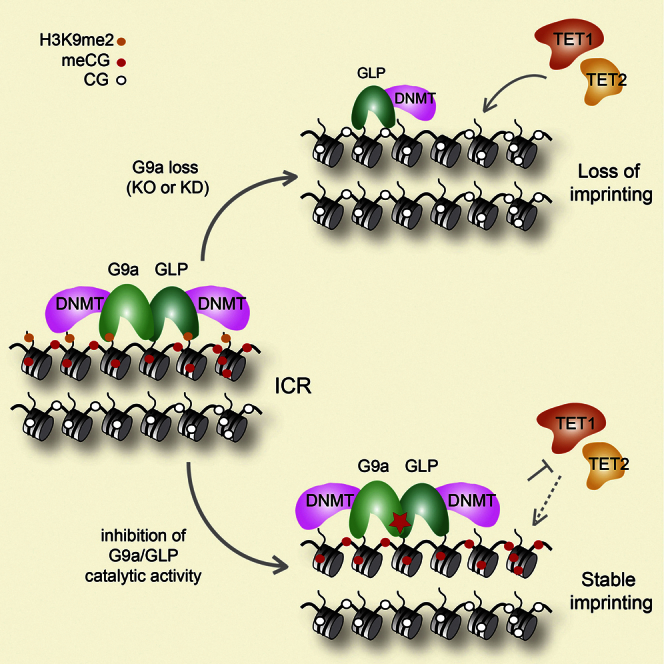

## Teaser

Loss of allele-specific DNA methylation from imprinting control regions leads to unbalanced gene expression and disease. Here, Zhang et al. show that the KMT enzymes G9a and GLP stabilize imprinted DNA methylation in embryonic stem cells by recruitment of de novo DNA methyltransferase enzymes, which counteract TET dioxygenase-dependent demethylation pathways.

## Introduction

Genomic imprinting is an epigenetic phenomenon, which ensures that certain genes are monoallelically expressed according to their parent of origin (Barlow and Bartolomei, 2014, Ferguson-Smith, 2011). In placental mammals, the imprinted genes regulate embryonic growth, brain functions, and energy homeostasis and tend to cluster at distinct chromosomal loci. The expression of imprinted genes is regulated in *cis* by imprinting control regions (ICRs), DNA sequences that acquire differential parent-specific DNA methylation during the maturation of male and female germ cells (Ferguson-Smith, 2011). Once established, the allele-specific DNA methylation at ICRs is stably maintained in the offspring through embryonic development and in somatic tissues. Loss of DNA methylation from ICRs leads to biallelic expression of imprinted genes and several human disorders associate with loss of imprinting and/or unbalanced expression of specific imprinted loci (Ferguson-Smith, 2011, Peters, 2014).

In mammals, the genome of the early zygote undergoes erasure of gamete-specific DNA methylation patterns in preparation for pluripotency and differentiation (Smith and Meissner, 2013). However, some sequences, among them the ICRs, escape the global reprogramming of DNA methylation (Smith et al., 2012, Wang et al., 2014) suggesting the existence of factors that protect these loci from erosion of DNA methylation. Recent studies identified several proteins that are required for stable maintenance of imprinted DNA methylation in the embryo and embryonic stem cells (ESCs). These include all DNA methyltransferases (DNMT1, DNMT3A, DNMT3B); the DNA and chromatin binding protein PGC7/STELLA; the Kruppel-associated box-containing zinc finger protein ZFP57 and its interacting partner KAP1/TRIM28 (Chen et al., 2003, Hirasawa et al., 2008, Li et al., 2008, Messerschmidt et al., 2012, Nakamura et al., 2007). The mechanism by which ZFP57 protects ICRs from loss of DNA methylation is attributed to sequence-specific binding of ZFP57 zinc fingers to methylated TGCCGC motif, present at most murine and some of the human ICRs, and recruitment of KAP1 together with histone H3 lysine 9 methylase SETDB1 and DNMTs. This complex promotes allelic maintenance of heterochromatin and DNA methylation at imprinted and some non-imprinted loci (Liu et al., 2012, Quenneville et al., 2011).

In addition to ZFP57, the histone H3 lysine 9 methylase G9a (EHMT2) and DNA/chromatin binding protein PGC7/STELLA are also implicated in maintenance of imprinted DNA methylation and protection of the maternal genome from TET dioxygenases-dependent DNA demethylation in early development (Nakamura et al., 2012). The maternal pronucleus in the zygote and the paternally methylated ICRs carry G9a-dependent H3 lysine 9 dimethylation (H3K9me2). This modification attracts PGC7/STELLA, which inhibits the action of TET enzymes at H3K9me2-marked heterochromatin. Such a model is consistent with the observed loss of DNA methylation from the maternal pronucleus and loss of imprinting in PGC7-null embryos (Nakamura et al., 2007). Whether maternally contributed G9a is required for maintenance of imprinted and non-imprinted DNA methylation in early embryos is yet to be determined.

G9a and the G9a-like protein GLP (EHMT1) form a G9a/GLP heterodimer in ESCs and function cooperatively to establish and maintain the abundant repressive H3K9me2 modification, in addition to modifying several non-histone proteins (Shinkai and Tachibana, 2011). The G9a-dependent H3K9me2 is implicated in lineage-specific gene silencing and covers large chromosomal domains associated with the nuclear lamina (Chen et al., 2012, Kind et al., 2013, Lienert et al., 2011). Disruption of either *G9a* or *Glp* genes in mice results in widespread loss of H3K9me2, growth retardation, and lethality of homozygous null embryos at E9.5–E10 (Tachibana et al., 2002, Tachibana et al., 2005). Importantly, the stability of G9a and GLP, particularly in embryonic stem cells (ESCs) and early embryos, is critically dependent on each other’s protein levels, providing an explanation for similarity of null phenotypes (Tachibana et al., 2005). Both G9a and GLP interact with DNMTs (Epsztejn-Litman et al., 2008, Estève et al., 2006), and loss of DNA methylation from repetitive sequences, specific non-imprinted loci, and from the maternally methylated *Snrpn* ICR was reported for *G9a*^*−/−*^ ESCs (Dong et al., 2008, Tachibana et al., 2008, Xin et al., 2003). Interestingly, expression of catalytically inactive G9a can partially restore DNA methylation, but not H3K9me2, in *G9a*^*−/−*^ ESCs (Dong et al., 2008, Tachibana et al., 2008) indicating that H3K9me2 is in part dispensable for G9a-dependent DNA methylation. Whether G9a, GLP, and H3K9me2 are required for DNA methylation at imprinted loci other than *Snrpn* has not been determined.

Here, we report that pluripotent stem cells null for either G9a or GLP display a widespread loss of imprinted DNA methylation, which can be reproduced by a small hairpin RNA (shRNA) knockdown of G9a in wild-type ESCs. Although the G9a-dependent H3K9me2 preferentially marks chromatin at methylated ICRs, we demonstrate that H3K9me2 is not essential for stable maintenance of imprinted DNA methylation in ESCs. Furthermore, we show that the G9a/GLP complex maintains imprinting by recruitment of de novo DNA methyltransferases, which antagonize the TET-enzyme-dependent DNA demethylation pathways.

## Results

### The *G9a*^*−/−*^ ESCs Display Widespread Loss of Imprinted DNA Methylation

**Figure 1:**
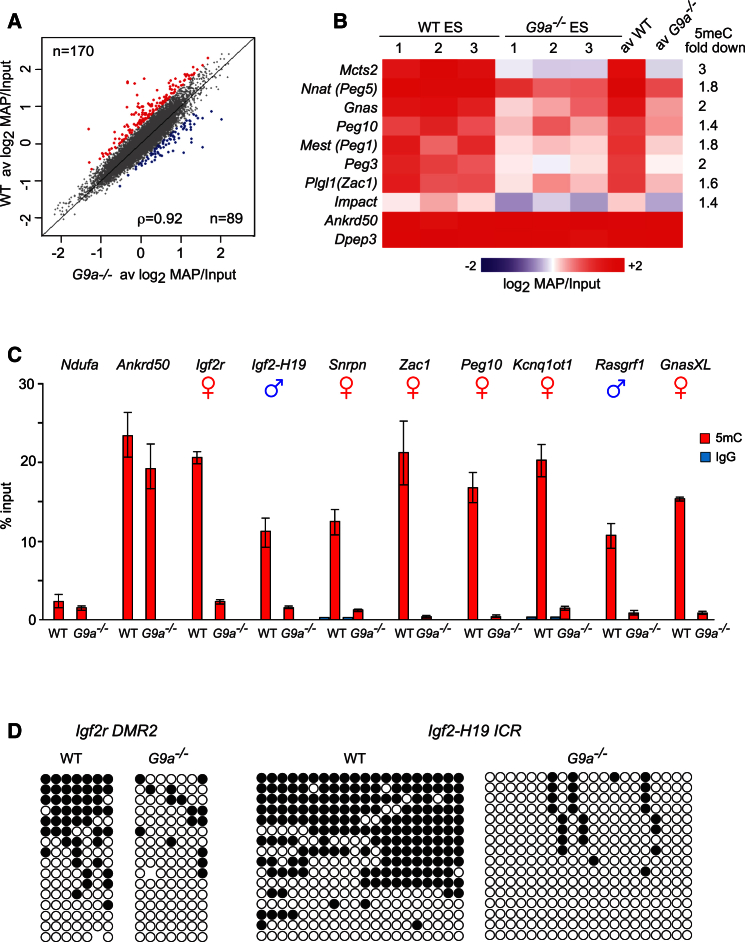
DNA Methylation Is Absent from ICRs in *G9a*^*−/−*^ ESCs (A) A log_2_ plot of the average DNA methylation at promoters of RefSeq genes in wild-type (WT) and *G9a*^***−/−***^ ESCs. Red and blue indicate promoters with ≥1.5-fold loss or gain of DNA methylation, respectively. (B) Heatmap of DNA methylation at maternally methylated promoter-associated ICRs and control regions (*Ankrd50* and *Dpep3*) in wild-type and *G9a*^***−/−***^ ESCs. Three biological replicate experiments are shown for each cell line as well as the average DNA methylation from the three experiments. (C) 5mC MeDIP followed by qPCR detects loss of imprinted DNA methylation from maternally and paternally methylated ICRs in *G9a*^***−/−***^ ESCs. Error bars represent SD, n = 3. See also Table S1. (D) Bisulfite DNA sequencing of *Igf2r* and *Igf2-H19* ICRs in wild-type and *G9a*^***−/−***^ ESCs. The black circles indicate methylated CpGs. See also Figure S1.

The *G9a*^***−/−***^ ESCs were reported to display global and locus-specific DNA hypomethylation (Dong et al., 2008, Estève et al., 2006, Myant et al., 2011). To examine whether the lack of G9a affects DNA methylation at promoters of protein coding genes, we previously employed methyl-CpG binding domain (MBD) affinity purification of methylated DNA (MAP) coupled with hybridization to promoter microarrays (Myant et al., 2011). These analyses identified 170 gene promoters that display reduced DNA methylation in *G9a*^***−/−***^ ESCs when compared to the parental wild-type ESCs (Figure 1A). Surprisingly, among the hypomethylated loci we identified eight promoters of maternally methylated imprinted genes, all of which represent germline ICRs (Figure 1B). To investigate further whether G9a deficiency affects all ICRs, including paternally methylated ICRs, we performed methylated DNA immunoprecipitation (MeDIP) on DNA from wild-type and *G9a*^***−/−***^ ESCs followed by qPCR. In all cases, we detected loss of DNA methylation from all of the examined ICRs (14 in total), while DNA methylation at control promoters, such as *Ankrd50*, was only mildly affected (Figure 1C; Table S1). Bisulfite DNA sequencing of five ICRs, including the maternally methylated *Igf2r DMR2*, *Mest*, *Snrpn*, *Kv DMR*, and the paternally methylated intergenic *H19-Igf2* ICR, as well as the control *Ankrd50* promoter was in agreement with the MeDIP data and detected normal imprinted DNA methylation in wild-type ESCs and nearly complete lack of DNA methylation at ICRs in *G9a*^***−/−***^ cells (Figures 1D and S1A). We observed a similar loss of DNA methylation in *Glp*^***−/−***^ ESCs (Figure S1B) indicating that both proteins, G9a and its interacting partner GLP, are required for normal patterns of imprinted DNA methylation at maternally and paternally methylated ICRs. Finally, the expression of wild-type G9a transgene in *G9a*^***−/−***^ ESCs did not restore DNA methylation at ICRs (Figure S1C). Taken together, these data reveal a widespread loss of imprinting in G9a- and GLP-null ESCs.

### Knockdown of G9a in ESCs Reduces DNA Methylation at ICRs

**Figure 2:**
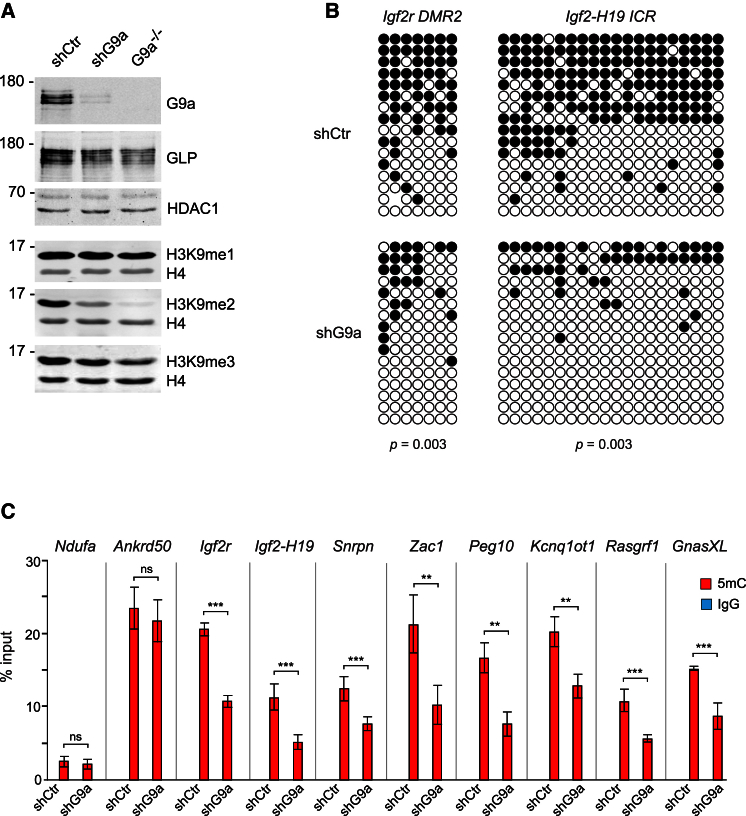
Knockdown of G9a in ESCs Leads to Loss of Imprinted DNA Methylation (A) Knockdown of G9a by small hairpin RNA (shG9a) reduces G9a, GLP, and H3K9me2 levels. shCrt is a non-silencing control shRNA. (B) Bisulfite DNA sequencing of *Igf2r* and *Igf2-H19* ICRs in shCtr and shG9a cell lines. (C) 5mC MeDIP followed by qPCR detects a decrease of DNA methylation at ICRs in shG9a ESCs. Error bars represent SD, n = 3, ^∗∗∗^p < 1e-3 (Mann-Whitney-Wilcoxon test). See also Figure S2 and Table S1.

Given that the *G9a*^***−/−***^ ESCs were generated by gene conversion from *G9a*^***+/−***^ ESCs (Tachibana et al., 2002) and that several reports have suggested that imprinting may become unstable upon long-term passaging of ESCs in culture (reviewed in Greenberg and Bourc’his, 2015), we asked whether the removal of G9a from wild-type ESCs would reproduce the loss of imprinted DNA methylation observed in the *G9a*^***−/−***^ ESCs. To investigate this, we stably knocked down G9a by small hairpin RNA (shG9a) in two wild-type ESC lines with different genetic background (TT2 and early passage E14) and generated clonal cell lines derived from single cells (Figures 2A and S2A). We also generated cells stably expressing a control shRNA (shCtr) that does not target any known murine RNA (Figure 2A). qRT-PCR and western blots detected a 70%–80% reduction of G9a mRNA and protein in cell lines with stably integrated shG9a plasmid (Figures 2A, S2A, and S2B). The G9a-dependent H3K9me2 was also significantly reduced in shG9a ESCs compared to controls while H3K9me1 and H3K9me3 remained largely unchanged (Figures 2A and S2B). Analyses of DNA methylation by bisulfite DNA sequencing and 5mC MeDIP detected significant loss of imprinted DNA methylation at all investigated ICRs in shG9a, but not in the shCtr ESCs (Figures 2B, 2C, and S2C; Table S1). These experiments demonstrate that both the knockout and the knockdown of G9a impair the stable maintenance of imprinted DNA methylation in ESCs.

### The Catalytic Activity of G9a and H3K9me2 Are Dispensable for Maintenance of Imprinted DNA Methylation

Potentially, either the G9a/GLP complex or its enzymatic methylase activity toward histone and non-histone substrates could be essential for stable maintenance of imprinted DNA methylation. About 90% of H3K9me2 in ESCs is dependent on G9a (see Figure 2A), and this heterochromatic modification may either directly or indirectly protect and stabilize DNA methylation at ICRs. The TET family dioxygenases (TET1 and TET2) are highly expressed in ESCs and have the ability to oxidize methylcytosine (5mC) to hydroxymethylcytosine (5hmC), which can serve as an intermediate toward unmethylated cytosine via active and passive demethylation pathways (Ito et al., 2010, Piccolo and Fisher, 2014). Thus, the G9a-dependent heterochromatin at methylated ICRs could potentially render these loci resistant to TET-dependent oxidation and further loss of 5mC. Alternatively, G9a/GLP could protect DNA methylation at ICR via an H3K9me2-independent mechanism.

**Figure 3:**
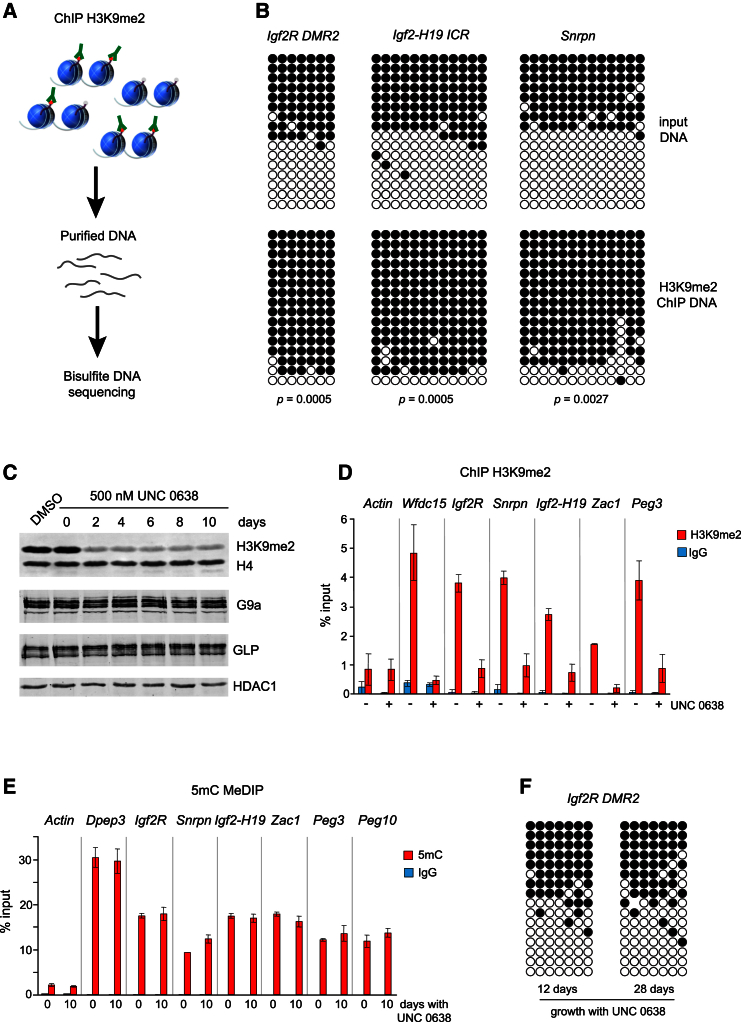
H3K9me2 Marks Preferentially Methylated ICRs but Is Dispensable for Maintenance of Imprinted DNA Methylation in ESCs (A) Schematic of ChIP followed by bisulfite DNA sequencing. (B) Bisulfite DNA sequencing of ICRs in input DNA and DNA purified from chromatin immunoprecipitated with anti-H3K9me2 antibody. p values were determined by chi-square test. (C) Treatment of cells with G9a/GLP inhibitor UNC 0638 leads to loss of H3K9me2, but does not affect the protein levels of G9a and GLP. (D) ChIP detects loss of H3K9me2 from ICRs in wild-type ESCs treated with UNC 0638. *Wfdc15* is a control non-imprinted methylated promoter marked by G9a-dependent H3K9me2 (Tachibana et al., 2008). Error bars represent SD, n = 3. (E) MeDIP analyses of ICRs in cells treated for 10 days with UNC 0638. Error bars represent SD, n = 3. (F) Bisulfite DNA sequencing of *Igf2r* ICR in cells treated with UNC 0638 for 12 and 28 days. See also Figure S3 and Table S1.

To address this, we first asked whether the G9a-dependent H3K9me2 was present specifically at methylated ICRs. Chromatin immunoprecipitation (ChIP) with anti-H3K9me2 antibodies from wild-type ESCs followed by bisulfite DNA sequencing revealed that, unlike in the input DNA, predominantly the methylated ICRs were present in the H3K9me2 antibody-precipitated chromatin (Figures 3A and 3B). Thus, H3K9me2 specifically marks the methylated copies of ICRs. To investigate further whether the G9a/GLP complex is responsible for H3K9me2 at methylated ICRs and whether or not H3K9me2 is required for maintenance of imprinted DNA methylation, we treated the wild-type ESCs with UNC 0638, a potent small molecule inhibitor of G9a and GLP catalytic activity (Vedadi et al., 2011). Although the treatment of ESCs with UNC 0638 led to a dramatic reduction of total and locus-specific H3K9me2 (Figures 3C and 3D), which was also accompanied by a global loss of 5mC (Figure S3A), the imprinted DNA methylation remained stable in UNC 0638-treated cells (Figures 3E and 3F; Table S1). Interestingly, the G9a complex as well as DNMT3A and DNMT3B remained stably associated with chromatin in cells treated with UNC 0638 (Figure S3B). From these experiments, we conclude that neither H3K9me2 nor the catalytic activity of G9a and GLP toward non-histone proteins is essential for maintenance of imprinted DNA methylation in ESCs.

### Recruitment of DNMTs via the ANK Domain of G9a Is Essential for Maintenance of Imprinted DNA Methylation

The experiments described above demonstrate that the G9a/GLP complex, but not its enzymatic activity, is required for maintenance of DNA methylation at ICRs. G9a was reported to interact directly, via its ankyrin repeat domain (ANK), with DNMT3A and DNMT3B and also indirectly, via GLP, with DNMT3A (Chang et al., 2011, Epsztejn-Litman et al., 2008). The N terminus of G9a was also shown to bind DNMT1 (Estève et al., 2006). To verify independently the association of G9a with DNMTs, we purified the G9a complex from ESCs and identified co-purifying proteins by mass spectrometry. These analyses revealed that DNMT3A, DNMT3B, DNMT3L, but not DNMT1, associate with the G9a complex in ESCs (Figures S3C and S3D). In addition to these interactions, an aromatic cage formed by a loop region between the fourth and the fifth ankyrin repeat within the ANK domain of G9a and GLP enables these enzymes to bind with micromolar affinity to H3K9me1 and H3K9me2 (Collins et al., 2008). Given these complex interactions, we hypothesized that the G9a/GLP complex could maintain the imprinted DNA methylation by promoting continuous recruitment of DNMTs to ICRs upon binding to the modified tails of histone H3.

**Figure 4:**
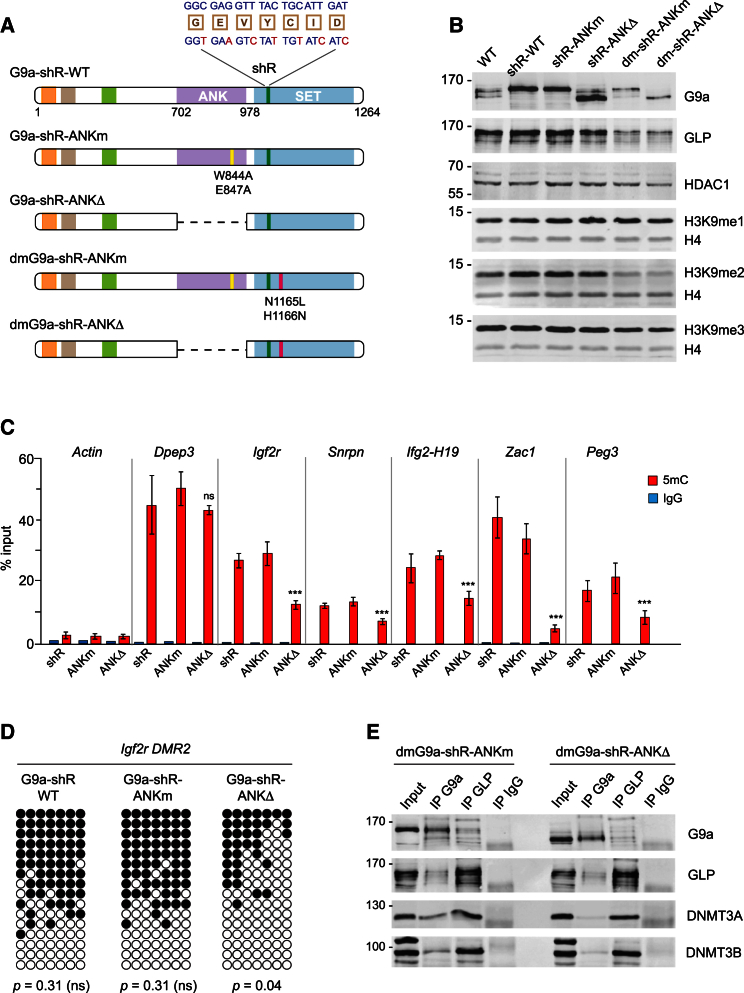
The ANK Domain of G9a and the Dimerization between G9a and GLP Are Required for Maintenance of Imprinted DNA Methylation (A) Schematic of shRNA-resistant (shR) wild-type and mutant forms of G9a. Silent mutations in shRNA targeted site (green) and point mutations disrupting either binding to H3K9me1/me2 (ANKm - yellow) or disrupting the dimerization of G9a with GLP (dm - red) are indicated. The dashed line represents ANK domain deletion (ANKΔ). (B) Protein levels of shRNA resistant wild-type and mutant G9a proteins in stable cell lines after knockdown of the endogenous G9a. Note that GLP and G9a are unstable, and H3K9me2 is reduced in cell lines expressing dimerization-deficient forms of G9a. (C) MeDIP detects reduced DNA methylation at ICRs in cells expressing G9a lacking the ANK domain (shR-ANKΔ). The error bars represent SD; n = 3; ^∗∗∗^p < 1e-3, ns p > 5e-2 (Mann-Whitney-Wilcoxon test). See also Table S1. (D) Bisulfite DNA sequencing of *Igf2r* ICR in cell lines expressing shR forms of G9a confirms the MeDIP data. p values were determined by chi-square test. (E) Detection of DNMTs by western blot in immunoprecipitations with anti-G9a or anti-GLP antibodies or mouse IgG from nuclear extracts of cells expressing dimerization-deficient G9a with either mutated (dm-shR-ANKm) or absent (dm-shR-ANKΔ) ANK domain. See also Figure S4.

To test this hypothesis, we generated ESC lines stably expressing shRNA-resistant either wild-type G9a (shR-WT) or mutant forms of G9a, which were either unable to bind H3K9me1/me2 (shR-ANKm) or lacked the entire ANK domain (shR-ANKΔ), respectively (Figure 4A). We then stably knocked down the endogenous G9a in these cell lines (Figure S4A) and examined H3K9me globally and DNA methylation at ICRs. All three cell lines (shR-WT, shR-ANKm, and sh-R-ANKΔ) displayed normal levels of H3K9me1, me2, and me3, which were indistinguishable from wild-type ESCs (Figure 4B). This indicates that the G9a ANK domain mutations and deletion do not impair significantly the binding of the G9a/GLP complex to chromatin, as binding could occur via the intact ANK domain of GLP. However, DNA methylation at ICRs was reduced in shR-ANKΔ, but not in shR-ANKm-expressing cells, as detected by MeDIP and bisulfite DNA sequencing (Figures 4C and 4D; Table S1). Consistent with the reported role of the ANK domain in binding DNMTs, we found that less DNMT3A and DNMT3B co-immunoprecipitated with shR-ANKΔ than with shR-ANKm G9a (Figures S4B and S4C). This could be observed more clearly in stable cell lines in which we replaced the endogenous G9a with mutant forms, either dm-shR-ANKm or dm-shR-ANKΔ, carrying additional point mutations (dm) that disrupted the dimerization of G9a with GLP (Figures 4A, 4B, and 4E). While GLP and dm-shR-ANKm still interacted with DNMTs, the dm-shR-ANKΔ G9a was unable to do so (Figure 4E). Since the formation of heterodimers stabilizes the G9a/GLP complex, G9a and GLP were unstable in cells expressing dimerization-deficient forms of G9a (Figure 4B), and both cell lines displayed loss of DNA methylation from ICRs (Figure S4D; Table S1). Collectively, these experiments demonstrate that the recruitment of DNMTs via the ANK domain of G9a and the formation of heterodimers between G9a and GLP are essential for stable maintenance of imprinted DNA methylation in ESCs.

### The Imprinted DNA Methylation Is Stable upon G9a Knockdown in TET-Deficient Cells

Two TET family enzymes, TET1 and TET2, are highly expressed in ESCs (Dawlaty et al., 2014) and may contribute to DNA methylation dynamics and heterogeneity, which are characteristic of ESCs grown in serum-containing medium (Shipony et al., 2014, Smallwood et al., 2014). Therefore, we hypothesized that the continuous recruitment of DNMTs to methylated ICRs by the G9a/GLP complex could counterbalance the action of TET enzymes and stabilize the imprinted DNA methylation in ESCs. If this were the case, then G9a/GLP would be dispensable for maintenance of imprinted DNA methylation in ESCs lacking TET enzymes.

**Figure 5:**
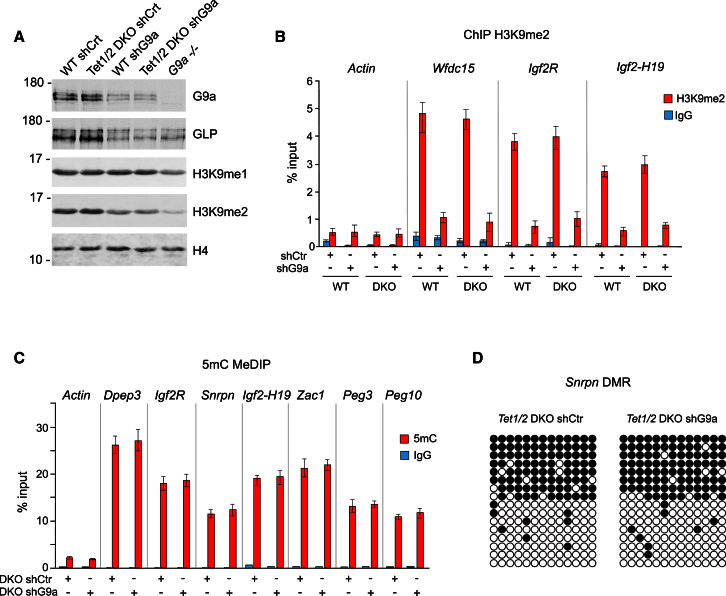
The Imprinted DNA Methylation Is Stable in G9a-Deficient Tet1/Tet2 Double-Knockout ESCs (A) Stable knockdown of G9a in wild-type and *Tet1/Tet2* double-knockout (DKO) ESCs by small hairpin RNA (shG9a). shCtr is a control non-targeting RNA. (B) ChIP experiments detect reduced H3K9me2 at ICRs in G9a-deficient wild-type and *Tet1/Tet2* DKO cells. Error bars represent SD, n = 3. See also Table S1. (C) 5mC MeDIP analyses show that DNA methylation at ICRs is stable in *Tet1/Tet2* DKO upon G9a knockdown. Error bars denote SD, n = 3. (D) Bisulfite DNA sequencing of *Snrpn* DMR in shCtr and shG9a *Tet1/Tet2* DKO ESCs. See also Figure S5.

To test this, we stably knocked down G9a in *Tet1*/*Tet2* double-knockout (DKO) ESCs (Dawlaty et al., 2014) (Figure 5A) and examined DNA methylation at ICRs by MeDIP and bisulfite DNA sequencing. The knockdown of G9a in *Tet1*/*Tet2* DKO cells and the decrease of G9a/GLP-dependent H3K9me2 were comparable between the *Tet1*/*Tet2* DKO and the control wild-type ESCs (Figures 5A and 5B; Table S1). However, unlike the shG9a ESCs expressing normal levels of TET enzymes (Figures 2B, 2C, and S2), DNA methylation at ICRs remained stable in *Tet1*/*Tet2* DKO shG9a cells and displayed patterns that were undistinguishable from the *Tet1*/*Tet2* DKO shCtr cells (Figures 5C and 5D). Moreover, the *Tet1/Tet2* DKO ESCs were also resistant to the UNC 0638-induced global loss of DNA methylation in comparison with their wild-type counterparts (Figure S5A). Together, these experiments demonstrate that the imprinted DNA methylation can be stably maintained in G9a-deficient ESCs if TET1 and TET2 enzymes are also absent. This suggests that the G9a/GLP-dependent recruitment of DNMTs to methylated ICRs stabilizes imprinting by antagonizing the activity of TET enzymes and TET-dependent 5mC demethylation pathways.

## Discussion

Stable maintenance of imprinted DNA methylation is important for ensuring that the allelic patterns established in gametes are preserved through the global reprogramming of 5mC in early development and in embryonic stem cells, which are characterized by a dynamic heterogeneity of DNA methylation (Shipony et al., 2014, Smallwood et al., 2014). Remarkably, the proteins implicated so far in maintenance of imprinted DNA methylation, such as ZFP57 and PGC7/STELLA, require the presence of pre-existing marks—either DNA or H3K9 methylation (Nakamura et al., 2012, Quenneville et al., 2011). Thus, the binding of ZFP57 to methylated DNA and recruitment of DNMTs via ZFP57-interacting proteins function as a self-reinforcing mechanism that ensures high local concentration of DNMTs and heterochromatin at methylated ICRs. On a more global scale, binding of PGC7/STELLA to G9a-dependent H3K9me2 was shown to protect the maternal genome as well as paternally methylated ICRs from TET-dependent 5mC hydroxylation and further conversion of 5hmC to unmethylated cytosine (Nakamura et al., 2012). Our data in part support these findings. The inhibition of G9a/GLP catalytic activity and the widespread depletion of H3K9me2 in UNC 0638-treated ESCs led to an ∼30% reduction of 5mC and a mild increase in 5hmC (Figure S5B). The loss of 5mC was largely TET1/TET2 dependent (Figure S5A) but did not affect the imprinted regions. In contrast, DNA methylation is reduced even further (∼40%) in *G9a*^***−/−***^ ESCs, and these cells are also characterized by significantly higher levels of 5hmC (Figure S5B). Consistently, both the knockout and the knockdown of G9a result in a widespread loss of DNA methylation from ICRs. Thus, although the G9a-dependent H3K9me2 preferentially marks methylated ICRs, H3K9me2 is dispensable for maintenance of DNA methylation at imprinted regions. Importantly, the loss of imprinted DNA methylation in G9a-deficient cells is also TET1/TET2 dependent, which is in agreement with the reported role of these proteins in the erasure of imprinted and not-imprinted DNA methylation in primordial germ cells in vivo (Yamaguchi et al., 2013). Collectively, these experiments demonstrate that the role of G9a/GLP in protecting the imprinted DNA methylation can be uncoupled from the catalytic activity of the complex and the contribution of H3K9me2 to maintenance of DNA methylation elsewhere in the genome. Additional experiments are required to map and compare on a wider scale the G9a- and H3K9me2-dependent loss of DNA methylation in ESCs and mouse embryos.

Notably, DNA methylation at specific loci, including promoters (Myant et al., 2011, Tachibana et al., 2008), satellite sequences, endogenous retrotransposons (Dong et al., 2008), and, as demonstrated here the methylated ICRs, is acutely dependent on G9a/GLP levels, but not on the enzymatic activity of the complex. Our experiments demonstrate that the loss of imprinted DNA methylation in *G9a*^***−/−***^ ESCs is not an artifact induced by the long-term passaging of the cells in culture as the stable knockdown of G9a in wild-type ESCs with normal imprinting also led to reduced DNA methylation at ICRs. Notably, the G9a knockout or knockdown did not affect the levels of ZFP57 in ESCs (data not shown) suggesting that G9a/GLP and ZFP57/KAP1 complexes may act cooperatively and reinforce each other’s function as neither complex on its own is sufficient to fully maintain the imprinted DNA methylation.

Our dissection of the H3K9me2-independent function of G9a in maintenance of imprinted DNA methylation led us to conclude that the ANK region, which was previously shown to interact with DNMT3A and DNMT3B (Epsztejn-Litman et al., 2008), as well the dimerization of G9a with GLP (Tachibana et al., 2008) are essential for DNA methylation at ICRs. Consistently, G9a lacking the ANK region was unable to interact with DNMTs and cells expressing G9a ANKΔ displayed reduced methylation at ICRs. These data strongly suggest that the continuous recruitment of DNMTs to methylated ICRs via the G9a/GLP and ZFP57/KAP1/SETDB1 complexes, rather than the repressive histone modifications established by their enzymatic activities, antagonize the action of TET enzymes and supports stable maintenance of imprinted DNA methylation in ESCs.

What attracts G9a/GLP complex to methylated ICR and sequences elsewhere in the genome is currently unknown and requires further investigation. Recruitment of G9a by non-coding RNA has been reported and might be important for the establishment and maintenance of DNA methylation and heterochromatin (Nagano et al., 2008, Skourti-Stathaki et al., 2014). Given that neither G9a nor GLP can bind RNA and/or DNA directly, such interactions are likely to involve additional components of the G9a/GLP complex, potentially the C2H2 zinc-finger proteins interacting with G9a/GLP (Maier et al., 2015, Shinkai and Tachibana, 2011) (Figure S3D). These proteins could bind either RNA or DNA in a sequence- and DNA methylation-specific manner and promote the recruitment of G9a/GLP complex to specific loci in the genome. Once H3K9me2 is established at such loci, the binding of G9a/GLP to H3K9me2 would allow spreading of H3K9me2 from the initial nucleation site to adjacent nucleosomes and stable maintenance of G9a/GLP binding and H3K9 and DNA methylation through successive cell divisions. Alternatively, it is possible that the unmethylated ICRs are protected from G9a binding and DNA methylation by the presence of refractive histone marks such as H3K4 and H3K27 di- and trimethylation (Henckel et al., 2009, McEwen and Ferguson-Smith, 2010).

Given that H3K9me2 is not essential for maintenance of imprinted DNA methylation in ESCs, it is likely that multiple mechanisms operate simultaneously to ensure that the G9a-dependent DNA and histone methylation at ICRs are preserved. It will be important to investigate further the role of G9a/GLP-interacting C2H2 zinc finger proteins and the largely overlooked role of H3K9me1 in the nucleation, spreading, and propagation of G9a-dependent DNA methylation and heterochromatin maintenance. Moreover, it will be essential to determine whether the maternally contributed and the zygote-expressed G9a and GLP stabilize and protect the imprinted DNA methylation in developing embryos.

## Experimental Procedures

### Cell Culture

Embryonic stem cells were grown in minimal essential medium (Life Technologies) supplemented with 10% fetal bovine serum (Thermo Scientific), non-essential amino acids, sodium pyruvate, β-mercaptoethanol, and leukemia inhibitory factor on gelatine-coated flasks (Greiner). Where indicated, the cells were grown in the presence of 500 nM of G9a/GLP inhibitor UNC 0638 (Sigma-Aldrich).

### Generation of Stable Cell Lines

Knockdown of G9a in ESCs and expression of mutant forms were performed by stable integration of electroporated plasmids. Detailed procedures are described in the Supplemental Information.

### Methylated DNA Affinity Purification and Promoter Microarrays

MAP of methylated DNA, hybridization to NimbleGen promoter microarrays, and data analyses were described previously (Myant et al., 2011). The data can be accessed at ArrayExpress: E-MEXP-2872.

### Methylated DNA Immunoprecipitation

Methylated DNA immunoprecipitation (MeDIP) was performed essentially as described (Weber et al., 2005) with anti-5mC antibody (Eurogentec) on sonicated genomic DNA. qPCRs were performed on 1/50 of immunoprecipitated DNA and 10 ng of input DNA. All MeDIP was carried out in three biological replicates with six technical replicates for each. Primers are listed in the Supplemental Information.

### Bisulfite DNA Sequencing

Genomic DNA was treated with EpiTect Bisulfite conversion kit (QIAGEN) and then used as a template for PCRs with specific primers listed in the Supplemental Information. PCR products were cloned into pJet vector (Thermo Scientific), sequenced using BigDye sequencing mix (Applied Biosystems) and analyzed by BiQ Analyzer software.

### Chromatin Immunoprecipitation

Chromatin immunoprecipitations (ChIPs) were performed as described previously (Myant et al., 2011) with anti-H3K9me2 (ab1220 Abcam; 07-441 Millipore) and non-specific mouse immunoglobulin G (IgG) (Sigma-Aldrich). Specific sequences were analyzed by qPCR on LC480 instrument (Roche). ChIP was performed in three biological replicates with six technical replicates for each.

### Western Blots

Nuclear proteins were extracted as in Myant et al. (2011), and specific proteins were analyzed as described in the Supplemental Information.

### Co-immunoprecipitations

Antibodies against G9a, GLP (R&D Systems Europe) and non-specific mouse IgG (Sigma-Aldrich) were used for immunoprecipitations from 400–500 μg of nuclear extract according to standard protocols. The amount of extract was doubled when dimerization-deficient forms of G9a were immunoprecipitated. The immunoprecipitated complexes and 1/10 of the input were resolved on 7% SDS-PAGE gels, transferred to nitrocellulose membranes, and detected as described above.

### Statistical Methods

Statistical methods used to analyze the promoter microarrays data (Figure 1A) were described in detail in Myant et al. (2011). Non-parametric two-tailed Mann-Whitney-Wilcoxon test was used to calculate significance (p values) for pairwise comparisons of qPCRs data following MeDIP (Figures 2C, 4C, S2C, S4D, and S5A). For analyses of bisulfite sequencing data (Figure 4D), the Chi-square test was applied to determine whether the differences between expected and observed methylation values were statistically significant. Standard parametric two-tailed t tests were used to calculate the p values for quantitative analyses of DNA methylation by reverse phase HPLC (Figures S3A, S5B, and S5C).

## Author Contributions

I.S. and T.Z. designed the project. T.Z. performed most experiments. A.T. carried out MBD affinity purifications, microarray analyses, and validation. X.X.B. helped with bisulfite DNA sequencing. J.C. and B.R. performed 5mC quantification. B.O. purified G9a-associated proteins. F.d.L.A. carried out mass spectrometry analyses. J.R. provided assistance with mass spectrometry. I.S., B.R., and T.Z. wrote the manuscript.

## Accession Numbers

The accession number for the proteomics data reported in this paper is PRIDE: PXD003466.

## Supplemental Information

Document S1. Supplemental Experimental Procedures and Figures S1–S5

Table S1. MeDIP and ChIP Data for Additional ICRs, Related to Figures 1C, 2C, 3D, 3E, 4C, S4D, and 5B

Document S2. Article plus Supplemental Information

## References

[bib1] Barlow D.P., Bartolomei M.S. (2014). Genomic imprinting in mammals. Cold Spring Harb. Perspect. Biol..

[bib2] Chang Y., Sun L., Kokura K., Horton J.R., Fukuda M., Espejo A., Izumi V., Koomen J.M., Bedford M.T., Zhang X. (2011). MPP8 mediates the interactions between DNA methyltransferase Dnmt3a and H3K9 methyltransferase GLP/G9a. Nat. Commun..

[bib3] Chen T., Ueda Y., Dodge J.E., Wang Z., Li E. (2003). Establishment and maintenance of genomic methylation patterns in mouse embryonic stem cells by Dnmt3a and Dnmt3b. Mol. Cell. Biol..

[bib4] Chen X., Skutt-Kakaria K., Davison J., Ou Y.L., Choi E., Malik P., Loeb K., Wood B., Georges G., Torok-Storb B., Paddison P.J. (2012). G9a/GLP-dependent histone H3K9me2 patterning during human hematopoietic stem cell lineage commitment. Genes Dev..

[bib5] Collins R.E., Northrop J.P., Horton J.R., Lee D.Y., Zhang X., Stallcup M.R., Cheng X. (2008). The ankyrin repeats of G9a and GLP histone methyltransferases are mono- and dimethyllysine binding modules. Nat. Struct. Mol. Biol..

[bib6] Dawlaty M.M., Breiling A., Le T., Barrasa M.I., Raddatz G., Gao Q., Powell B.E., Cheng A.W., Faull K.F., Lyko F., Jaenisch R. (2014). Loss of Tet enzymes compromises proper differentiation of embryonic stem cells. Dev. Cell.

[bib7] Dong K.B., Maksakova I.A., Mohn F., Leung D., Appanah R., Lee S., Yang H.W., Lam L.L., Mager D.L., Schübeler D. (2008). DNA methylation in ES cells requires the lysine methyltransferase G9a but not its catalytic activity. EMBO J..

[bib8] Epsztejn-Litman S., Feldman N., Abu-Remaileh M., Shufaro Y., Gerson A., Ueda J., Deplus R., Fuks F., Shinkai Y., Cedar H., Bergman Y. (2008). De novo DNA methylation promoted by G9a prevents reprogramming of embryonically silenced genes. Nat. Struct. Mol. Biol..

[bib9] Estève P.O., Chin H.G., Smallwood A., Feehery G.R., Gangisetty O., Karpf A.R., Carey M.F., Pradhan S. (2006). Direct interaction between DNMT1 and G9a coordinates DNA and histone methylation during replication. Genes Dev..

[bib10] Ferguson-Smith A.C. (2011). Genomic imprinting: the emergence of an epigenetic paradigm. Nat. Rev. Genet..

[bib11] Greenberg M.V., Bourc’his D. (2015). Cultural relativism: maintenance of genomic imprints in pluripotent stem cell culture systems. Curr. Opin. Genet. Dev..

[bib12] Henckel A., Nakabayashi K., Sanz L.A., Feil R., Hata K., Arnaud P. (2009). Histone methylation is mechanistically linked to DNA methylation at imprinting control regions in mammals. Hum. Mol. Genet..

[bib13] Hirasawa R., Chiba H., Kaneda M., Tajima S., Li E., Jaenisch R., Sasaki H. (2008). Maternal and zygotic Dnmt1 are necessary and sufficient for the maintenance of DNA methylation imprints during preimplantation development. Genes Dev..

[bib14] Ito S., D’Alessio A.C., Taranova O.V., Hong K., Sowers L.C., Zhang Y. (2010). Role of Tet proteins in 5mC to 5hmC conversion, ES-cell self-renewal and inner cell mass specification. Nature.

[bib15] Kind J., Pagie L., Ortabozkoyun H., Boyle S., de Vries S.S., Janssen H., Amendola M., Nolen L.D., Bickmore W.A., van Steensel B. (2013). Single-cell dynamics of genome-nuclear lamina interactions. Cell.

[bib16] Li X., Ito M., Zhou F., Youngson N., Zuo X., Leder P., Ferguson-Smith A.C. (2008). A maternal-zygotic effect gene, Zfp57, maintains both maternal and paternal imprints. Dev. Cell.

[bib17] Lienert F., Mohn F., Tiwari V.K., Baubec T., Roloff T.C., Gaidatzis D., Stadler M.B., Schübeler D. (2011). Genomic prevalence of heterochromatic H3K9me2 and transcription do not discriminate pluripotent from terminally differentiated cells. PLoS Genet..

[bib18] Liu Y., Toh H., Sasaki H., Zhang X., Cheng X. (2012). An atomic model of Zfp57 recognition of CpG methylation within a specific DNA sequence. Genes Dev..

[bib19] Maier V.K., Feeney C.M., Taylor J.E., Creech A.L., Qiao J.W., Szanto A., Das P.P., Chevrier N., Cifuentes-Rojas C., Orkin S.H. (2015). Functional Proteomic Analysis of Repressive Histone Methyltransferase Complexes Reveals ZNF518B as a G9A Regulator. Mol. Cell. Proteomics.

[bib20] McEwen K.R., Ferguson-Smith A.C. (2010). Distinguishing epigenetic marks of developmental and imprinting regulation. Epigenetics Chromatin.

[bib21] Messerschmidt D.M., de Vries W., Ito M., Solter D., Ferguson-Smith A., Knowles B.B. (2012). Trim28 is required for epigenetic stability during mouse oocyte to embryo transition. Science.

[bib22] Myant K., Termanis A., Sundaram A.Y., Boe T., Li C., Merusi C., Burrage J., de Las Heras J.I., Stancheva I. (2011). LSH and G9a/GLP complex are required for developmentally programmed DNA methylation. Genome Res..

[bib23] Nagano T., Mitchell J.A., Sanz L.A., Pauler F.M., Ferguson-Smith A.C., Feil R., Fraser P. (2008). The Air noncoding RNA epigenetically silences transcription by targeting G9a to chromatin. Science.

[bib24] Nakamura T., Arai Y., Umehara H., Masuhara M., Kimura T., Taniguchi H., Sekimoto T., Ikawa M., Yoneda Y., Okabe M. (2007). PGC7/Stella protects against DNA demethylation in early embryogenesis. Nat. Cell Biol..

[bib25] Nakamura T., Liu Y.J., Nakashima H., Umehara H., Inoue K., Matoba S., Tachibana M., Ogura A., Shinkai Y., Nakano T. (2012). PGC7 binds histone H3K9me2 to protect against conversion of 5mC to 5hmC in early embryos. Nature.

[bib26] Peters J. (2014). The role of genomic imprinting in biology and disease: an expanding view. Nat. Rev. Genet..

[bib27] Piccolo F.M., Fisher A.G. (2014). Getting rid of DNA methylation. Trends Cell Biol..

[bib28] Quenneville S., Verde G., Corsinotti A., Kapopoulou A., Jakobsson J., Offner S., Baglivo I., Pedone P.V., Grimaldi G., Riccio A., Trono D. (2011). In embryonic stem cells, ZFP57/KAP1 recognize a methylated hexanucleotide to affect chromatin and DNA methylation of imprinting control regions. Mol. Cell.

[bib29] Shinkai Y., Tachibana M. (2011). H3K9 methyltransferase G9a and the related molecule GLP. Genes Dev..

[bib30] Shipony Z., Mukamel Z., Cohen N.M., Landan G., Chomsky E., Zeliger S.R., Fried Y.C., Ainbinder E., Friedman N., Tanay A. (2014). Dynamic and static maintenance of epigenetic memory in pluripotent and somatic cells. Nature.

[bib31] Skourti-Stathaki K., Kamieniarz-Gdula K., Proudfoot N.J. (2014). R-loops induce repressive chromatin marks over mammalian gene terminators. Nature.

[bib32] Smallwood S.A., Lee H.J., Angermueller C., Krueger F., Saadeh H., Peat J., Andrews S.R., Stegle O., Reik W., Kelsey G. (2014). Single-cell genome-wide bisulfite sequencing for assessing epigenetic heterogeneity. Nat. Methods.

[bib33] Smith Z.D., Meissner A. (2013). DNA methylation: roles in mammalian development. Nat. Rev. Genet..

[bib34] Smith Z.D., Chan M.M., Mikkelsen T.S., Gu H., Gnirke A., Regev A., Meissner A. (2012). A unique regulatory phase of DNA methylation in the early mammalian embryo. Nature.

[bib35] Tachibana M., Sugimoto K., Nozaki M., Ueda J., Ohta T., Ohki M., Fukuda M., Takeda N., Niida H., Kato H., Shinkai Y. (2002). G9a histone methyltransferase plays a dominant role in euchromatic histone H3 lysine 9 methylation and is essential for early embryogenesis. Genes Dev..

[bib36] Tachibana M., Ueda J., Fukuda M., Takeda N., Ohta T., Iwanari H., Sakihama T., Kodama T., Hamakubo T., Shinkai Y. (2005). Histone methyltransferases G9a and GLP form heteromeric complexes and are both crucial for methylation of euchromatin at H3-K9. Genes Dev..

[bib37] Tachibana M., Matsumura Y., Fukuda M., Kimura H., Shinkai Y. (2008). G9a/GLP complexes independently mediate H3K9 and DNA methylation to silence transcription. EMBO J..

[bib38] Vedadi M., Barsyte-Lovejoy D., Liu F., Rival-Gervier S., Allali-Hassani A., Labrie V., Wigle T.J., Dimaggio P.A., Wasney G.A., Siarheyeva A. (2011). A chemical probe selectively inhibits G9a and GLP methyltransferase activity in cells. Nat. Chem. Biol..

[bib39] Wang L., Zhang J., Duan J., Gao X., Zhu W., Lu X., Yang L., Zhang J., Li G., Ci W. (2014). Programming and inheritance of parental DNA methylomes in mammals. Cell.

[bib40] Weber M., Davies J.J., Wittig D., Oakeley E.J., Haase M., Lam W.L., Schübeler D. (2005). Chromosome-wide and promoter-specific analyses identify sites of differential DNA methylation in normal and transformed human cells. Nat. Genet..

[bib41] Xin Z., Tachibana M., Guggiari M., Heard E., Shinkai Y., Wagstaff J. (2003). Role of histone methyltransferase G9a in CpG methylation of the Prader-Willi syndrome imprinting center. J. Biol. Chem..

[bib42] Yamaguchi S., Shen L., Liu Y., Sendler D., Zhang Y. (2013). Role of Tet1 in erasure of genomic imprinting. Nature.

