# Supplementary material for: G9a/GLP Complex Maintains Imprinted DNA Methylation in Embryonic Stem Cells
Source: Cell Rep. 2016 Mar 24;15(1):77–85. doi: 10.1016/j.celrep.2016.03.007 (PMC4826439; doi:10.1016/j.celrep.2016.03.007)
Supplement: Document S1. Supplemental Experimental Procedures and Figures S1–S5 [file mmc1.pdf]

Cell Reports, Volume *15*

Supplemental Information

G9a/GLP Complex Maintains Imprinted DNA Methylation in Embryonic Stem Cells

Tuo Zhang, Ausma Termanis, Burak Özkan, Xun X. Bao, Jayne Culley, Flavia de Lima Alves, Juri Rappsilber, Bernard Ramsahoye, and Irina Stancheva

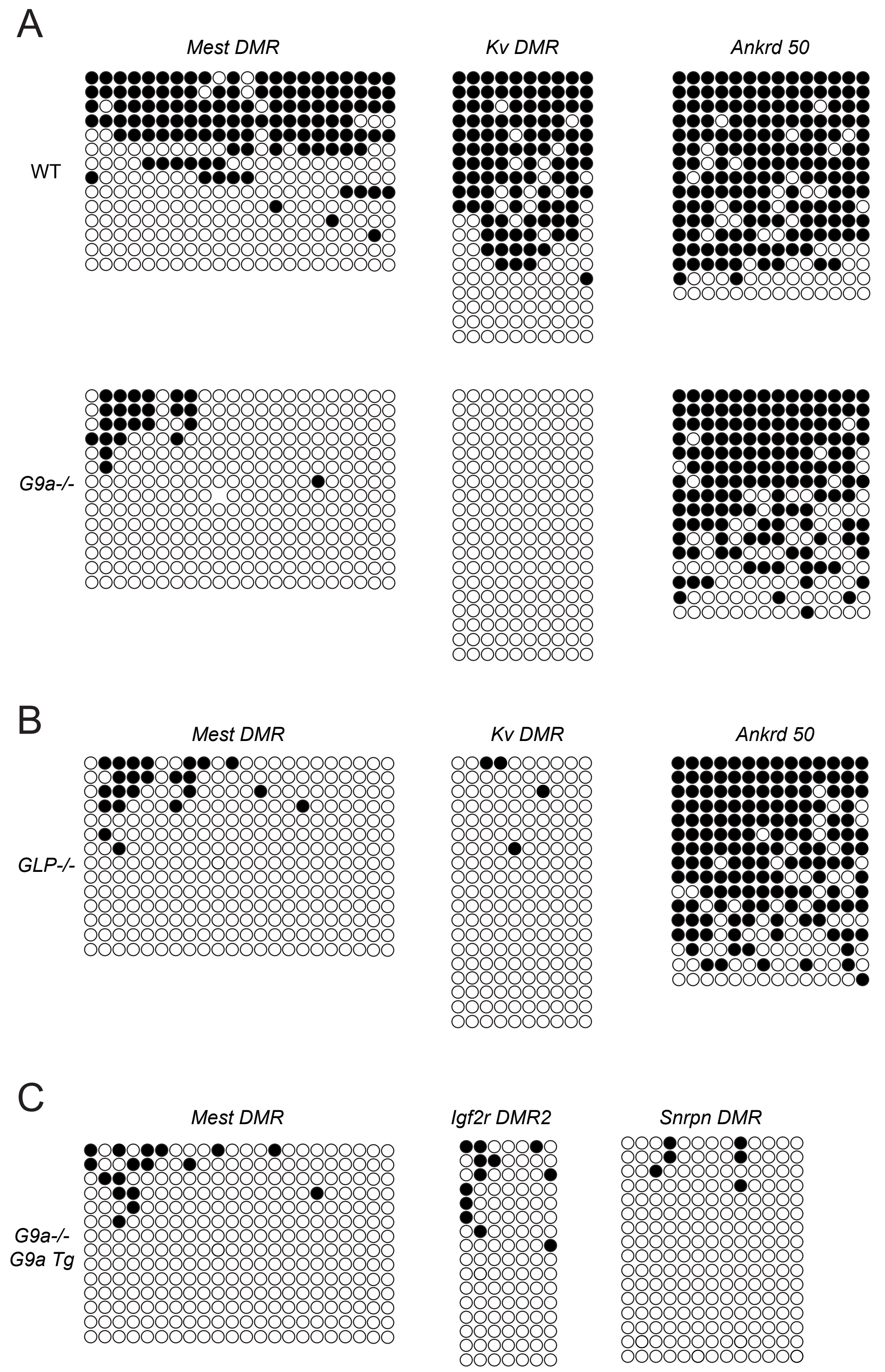

Figure S1 (related to Figure 1) DNA methylation is absent from ICRs in *G9a-/-*, *Glp-/-* and *G9a-/- G9a Tg* ESCs
A. Bisulfite DNA sequencing of maternally methylated *Mest* and *Kv* ICRs in wild-type and *G9a-/-* ESCs. *Ankrd 50* is a control non-imprinted methylated promoter the methylation of which is not significantly affected by G9a deficiency. **B.** Bisulfite DNA sequencing of *Mest* and *Kv* ICRs as well as *Ankrd 50* promoter in *Glp-/-* ESCs. **C.** Stable expression of wild-type G9a transgene does not restore the imprinted DNA methylation in *G9a-/-* ESCs.

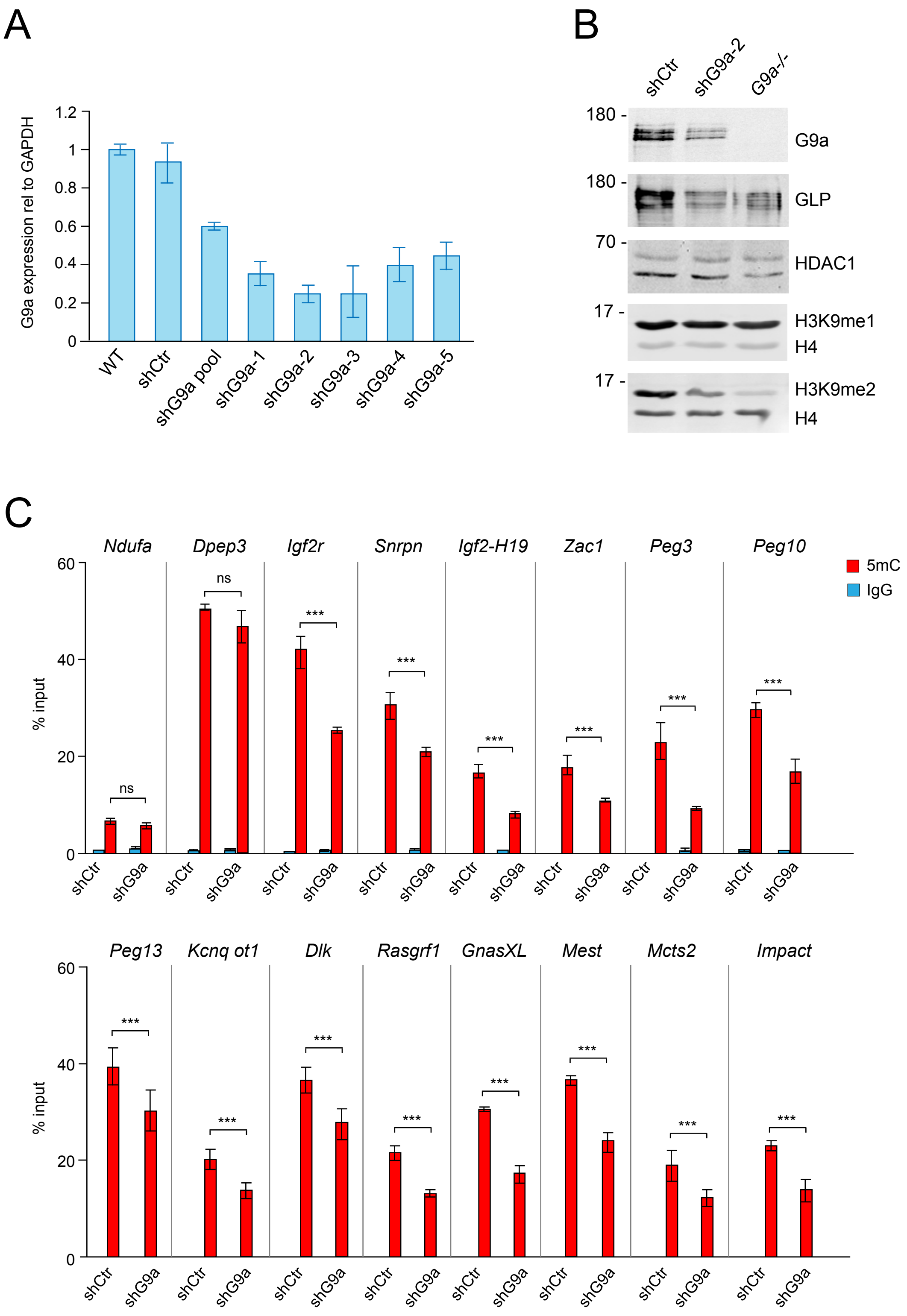

Figure S2 (related to Figure 2) Knockdown of G9a in E14 ESCs also leads to loss of DNA methylation from imprinted loci

A. G9a mRNA levels in clonal shG9a knockdown E14 ES cell lines as assessed by quantitative RT-PCR. The cell line with lowest levels of G9a transcript, shG9a-2, was used for further analyses. **B.** Quantitative Western blots detect significantly reduced levels of G9a protein (~10% of WT levels) and H9K9me2 (~18% of WT levels) in shG9a E14 ESCs. H3K9me1 is reduced by 10% in shG9a cells and by 15% in *G9a-/-* ESCs. **C.** The knockdown of G9a in E14 ESCs results in reduced DNA methylation at imprinted loci as detected by MeDIP with anti-5mC antibodies. An anti-mouse IgG was used as a control. *Ndufa* and *Dpep3* are control unmethylated and methylated promoters, respectively. The error bars represent standard deviation, n=3. *** p<1e-3 and ns p>5e-2 (Wilcoxon-Mann-Whitney test).

A

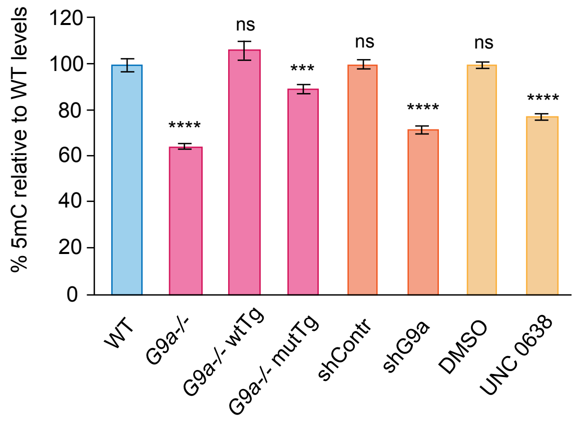

C

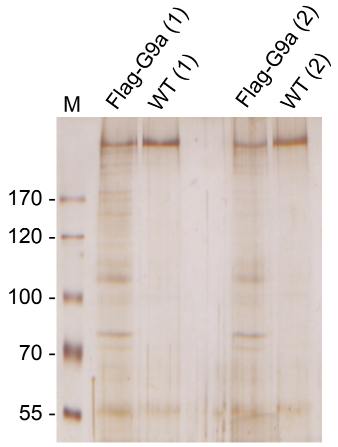

B

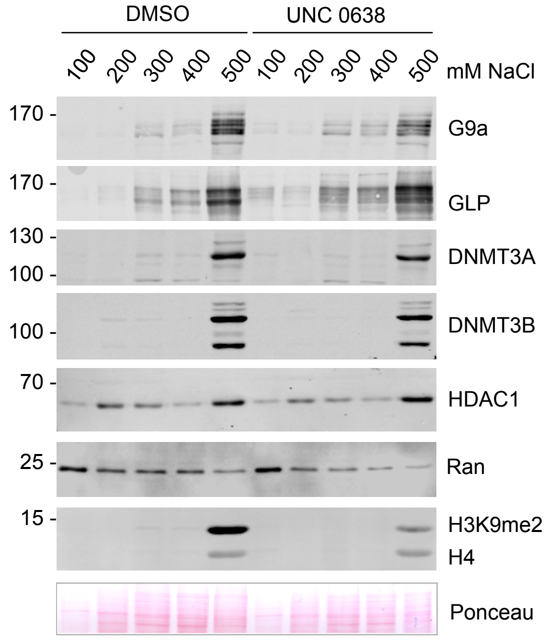

D

No. peptides	Flag-G9a (1)	WT (1)	Flag-G9a (2)	WT (2)	
G9a (EHMT2)	11	0	17	0	core complex
GLP (EHMT1)	13	0	15	0	
WIZ	12	0	12	0	
ZFP462	23	0	24	0	
DNMT3L	8	0	8	0	
DNMT3A	4	1	2	0	
DNMT3B	2	0	6	0	
LSH (HELLS)	7	0	5	0	
CBP	5	0	5	0	
ZFP518B	5	0	5	0	

Figure S3 (related to Figure 3) G9a-dependent DNA methylation, the association of G9a with chromatin and with other proteins

A. Quantitative analyses of total 5mC by high performance liquid chromatography (HPLC) in genomic DNA purified from wild-type (TT2) ESCs, *G9a-/- ESCs*, *G9a-/-* ESCs carrying either wild-type (wtTg) or catalytically-inactive (mutTg) G9a transgene, cells expressing a control small hairpin RNA (shConrt), G9a mRNA-targeting small hairpin RNA (shG9a), and ESCs treated with either DMSO or G9a/GLP inhibitor UNC0638 for 6 days. The error bars represent standard deviation and the symbols above each bar are two-tailed *p* values (t-test) calculated for pairwise comparison with the wild-type cells; n=3 ****p<1e-4, *** is p<1e-3, **p<1e-2 and ns (not significant) p>5e-2 **B.** Fractions from a step-wise extraction of nuclear proteins from wild-type ESCs grown either in the presence of DMSO or the G9a/GLP inhibitor UNC 0638 for 14 days. The fractions (equal volume of each) were run on SDS gels, blotted and the membranes probed with the indicated antibodies. G9a, GLP and DNMTs stably associate with chromatin both in the control (DMSO) and in the inhibitor-treated cells. These experiments indicate that H3K9me2 is not essential for binding of G9a/GLP and DNMTs to chromatin/DNA. **C.** Silver stained gel of Flag affinity purified proteins from either *G9a-/-* ESCs stably expressing Flag-G9a or from wild-type ESCs that express untagged G9a. Two independent purification (1) and (2) are shown. **D.** Mass spectrometry analyses of the samples shown in (C) identified proteins that co-purify with Flag-G9a, including the core components of the G9a complex (GLP, WIZ), known G9a associated proteins (CBP), zinc finger proteins *de novo* DNMT and their associated factors DNMT3L and LSH. Only a proportion of the identified proteins is shown in the table.

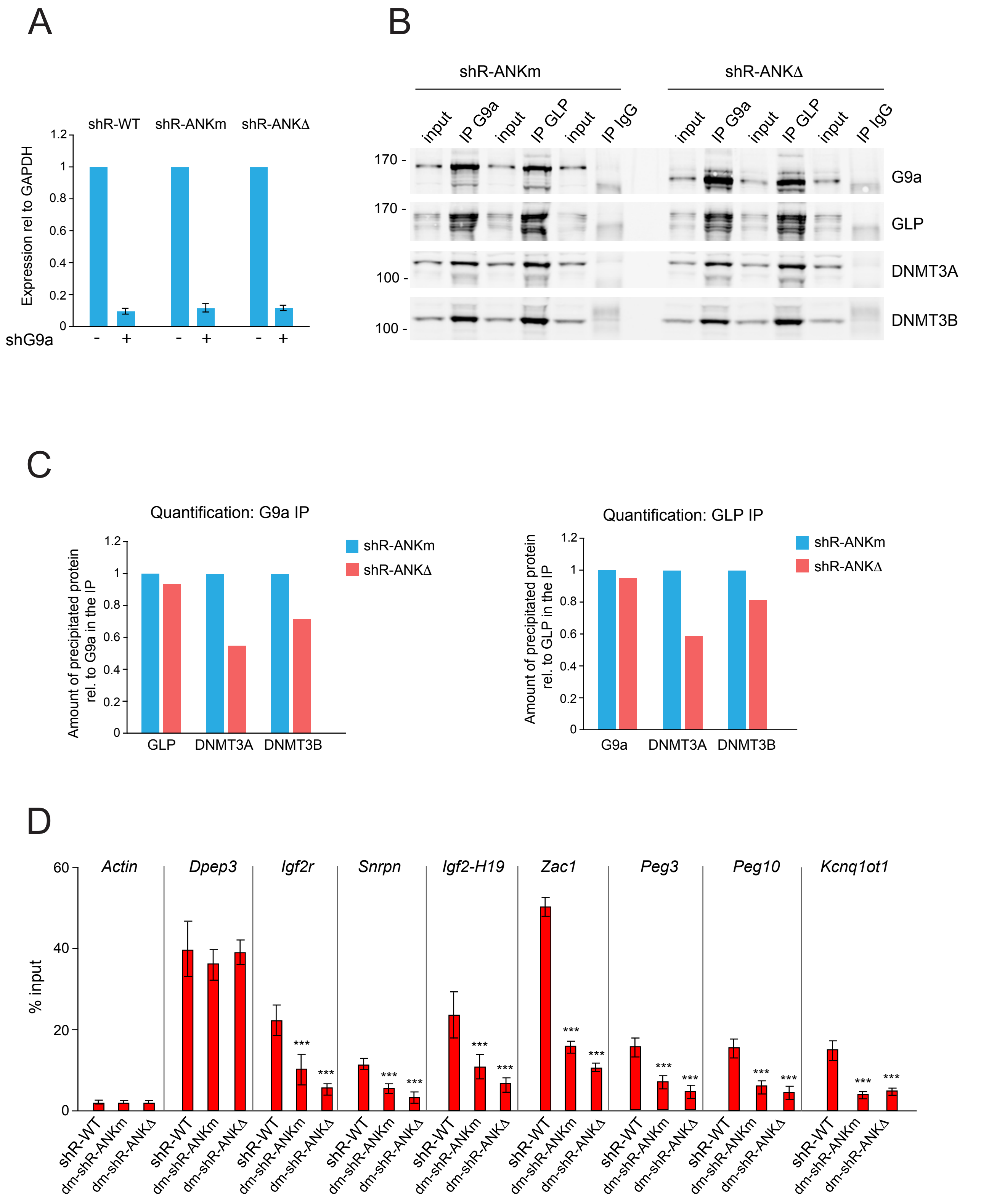

Figure S4 (related to Figure 4) Characterization of ES cell lines expressing mutant forms of G9a

A. Quantitative RT-PCR analyses confirm the successful knockdown of the endogenous G9a in ES cell lines expressing exogenous shG9a-resistant forms of G9a: wild-type (shR-WT), G9a with point mutations in the ANK domain (shR-ANKm) and G9a with deleted ANK domain (shR-ANKΔ). **B.** Mutant forms of G9a (shR-ANKm and shR-ANKΔ) as well as GLP co-imunoprecipitate with DNMTs. **C.** Quantification of protein amounts co-immuno-precipitated with G9a (left) and GLP (right) from cells expressing either shR-ANKm or shR-ANKΔ. Note that the anti-G9a antibodies immunoprecipitate less DNMTA and DNMTB from cells expressing shR-ANKΔ G9a.
D. DNA methylation at ICRs as examined by MeDIP is reduced in ESCs expressing dimerization-deficient forms of G9a. The error bars in **A.** and **C.** represent standard deviation, n=3 *** p<1e-3 (Mann-Whitney-Wilcoxon test) for comparisons between shR-WT and dimerisation-deficient (dm) shR mutant forms of G9a.

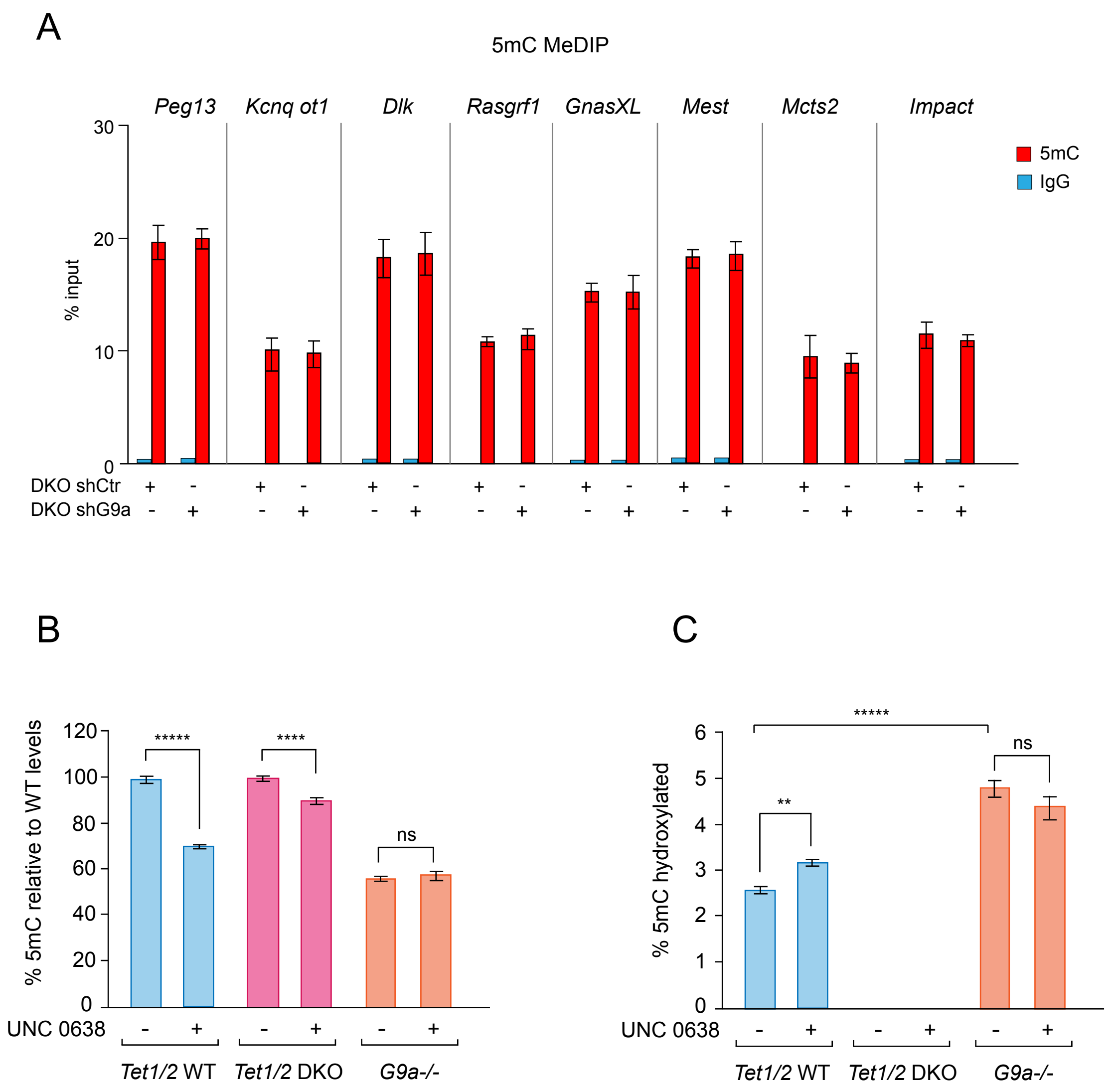

Figure S5 (related to Figure 5) The *Tet1/Tet2* DKO cells are resistant to G9a knockdown and G9a/GLP inhibitor-induced DNA hypomethylation

A. Analyses of DNA methylation by MeDIP at eight additional ICRs in shCtr and shG9a *Tet1/Tet2* DKO ESCs. The error bars represent standard deviation; n=3; *p*>5e-2 for all pairwise comparisons (Mann-Whitney-Wilcoxon test). **B.** Quantitative analyses of total 5mC by high performance liquid chromatography (HPLC) in genomic DNA purified from wild-type, *Tet1/Tet*2 DKO and *G9a-/- ESCs*, *G9a-/-* ESCs that were grown either in the presence of DMSO (-) or G9a/GLP inhibitor UNC 0638 (500nM) for 14 days (+). The error bars represent standard deviation, n=3. The stars above the graph are *p* values (t-test): ***** *p* < 1e-5, **** *p* < 1e-4, ns *p*>5e-2 **C.** Quantification of hydroxylated 5mC as a percentage of total 5mC in wild-type and *G9a-/-* ES cells grown with either DMSO (-) or G9a/GLP inhibitor UNC 0638 (+) for 14 days. Hydroxylated 5mC was not detectable in *Tet1/Tet2* DKO cells. Error bars represent standard deviation; n=3; *p* values (t) test ***** *p*>1e-5, ** *p* <1e-2; ns *p*>5e-2

Supplemental Experimental Procedures

Generation of stable cell lines

Sure Silencing plasmids (QIAGEN) expressing either control or G9a-targeting small hairpin RNA and carrying either hygromycin or puromycin resistance marker were introduced into ESCs by electroporation. The electroporated cells were plated at low density on gelatine-coated plates and grown under selection with 2 μg/ml puromycin or 100 μg/ml hygromycin until single cell-derived colonies formed. Individual colonies were transferred into 6-well plates and expanded into clonal cell lines. The efficiency of G9a knockdown was determined by quantitative reverse transcription PCR and Western blots. Mutant versions of G9a cDNA were generated by PCR, cloned into a pCAG-puro plasmid and stably integrated into ESCs as described above prior to knocking down the endogenous G9a. Primers to test the efficiency of G9a knockdown in cells expressing shRNA-resistant mutant forms of G9a spanned the shRNA target sequence (see List of primers).

Western blots

Nuclear proteins were extracted as described in Myant et al, 2011, resolved in either 7% or 15% SDS-PAGE gels and transferred to nitrocellulose (non-histone proteins) or PVDF (histones) membranes (BioRad). The membranes were blocked with 4% skimmed milk in 1xTBS buffer supplemented with 0.1% Tween-20 and incubated with primary antibodies overnight and secondary either IR800 or IR670L labelled antibodies (LI-COR Biosciences) for 1 hour. The signals were detected on Odyssey IR scanner (LI-COR Biosciences) and quantified where appropriate with LO-COR Image Studio software. The primary antibodies are listed in Supplemental Methods.

Analyses of DNA methylation by reverse-phase HPLC

DNA was extracted from ESCs following standard protocols and residual RNA was removed by enzymatic hydrolysis (6-hour incubation with RNase A and RNase T1) followed by DNA precipitation in 3 volumes of ethanol. This was repeated once. 1-5 ug of purified DNA was digested with DNase1 (New England Biolabs) for 12 hours in the recommended buffer. Following this, 2 volumes of 30 mM sodium acetate pH 5.2 was added and the DNA was further digested to nucleotide 5' monophosphates with Nuclease P1 (Sigma) in the presence of 1 mM zinc sulphate (7 hour incubation). The Quantitation of 5-methylcytosine in genomic DNA was performed in triplicate by isocratic high performance reverse phase liquid chromatography as previously described (Ramsahoye, 2002), with the following alterations. A Dionex UM 3000 HPLC system was used complete with a column chiller, C18 column (250mm x 4.6 mm 5 um APEX ODS, #4M25310, Grace Discovery Sciences), and column guard (Phomenex, #AJ0-7596). The mobile phase was 50 mM ammomium phosphate (monobasic) pH4.1, flow rate 1

ml/min. The column was chilled to 8°C to improve peak separation. Deoxyribonucleotides (dNMPs) were detected at their extinction maxima using a Dionex 3000 multiple wavelength detector: dCMP, 276 nm, retention time 9 minutes; 5hmdCMP, 282 nm, retention time 10.5 minutes; dCMP, 282 nm, retention time 16.5 minutes. Nucleotide quantifications were calculated from the area under each peak using Chromeleon software and using the respective extinction coefficients (dCMP, 8.86 x 10^3; 5hmdCMP, 7.7 x 10^3; 5mdCMP 9.0 x 10^3)

Purification of the G9a complex

Nuclear extracts were prepared using standard protocols from wild-type (TT2) ESCs and *G9a-/-* ESCs stably expressing Flag-G9a. Briefly, the cells were disrupted in hypotonic NE1 buffer (20% glycerol, 20 mM HEPES, pH 7; 10 mM KCl, 1 mM $MgCl_2$, 0.5 mM DTT, 0.1% Triton) and the nuclei collected by centrifugation at 4000 rpm for 5min at 4°C. The nuclei were digested with 100u of Benzonase nuclease (Merk) in NE1 and nuclear proteins were extracted by homogenising the nuclei in NE1 containing 300 mM NaCl and incubation at 4°C for 1h on a rotating wheel. The suspension was centrifuged at 13000 rpm for 15 min and the soluble proteins separated from the pellet containing insoluble proteins and membranes. 750 μg of total nuclear protein from each cell line were incubated with 25 ml FLAG® M2 agarose beads (Sigma) for 3 hours at 4°C on a rotating wheel. The beads were pelleted for 1 min at 4000 rpm and washed 4 times with NE1 buffer containing 200 mM NaCl. After the last wash the bound proteins were eluted from the beads overnight at 4°C with 100 ml NE1 buffer containing 200 mM NaCl, 5 mM DTT and 70 μg of FLAG peptide (Sigma). The eluted proteins were collected by passing the bead suspensions through mini-columns (C2603-200EA, Sigma) and analysed further on 7% SDS-polyacrylamide gels and by mass spectrometry. The data can be accessed at proteomics database PRIDE under accession number PXD003466.

Step-wise extraction of nuclear proteins

2x10^6 cells were used in each extraction experiment. The cells were lysed in 1 ml hypotonic NE1 buffer (see above) and the nuclei pelleted at 4000 rpm for 5 min at 4°C. In each subsequent extraction step, the nuclei were resuspended in 150 ml NE1 buffer supplemented with increasing concentrations of NaCl (100, 200, 300, 400 mM), incubated for 30 min on rotating wheel at 4°C and spun down at 4000 rpm for 5 min at 4°C before adding the next buffer. At the last step, the nuclei were washed with NE1, digested with 100u of Benzonase nuclease and the chromatin-associated proteins were extracted with 500 mM NaCl.

Mass spectrometry analyses

Protein samples were run on 10% SDS-PAGE gel, for 5 min, stained with coomassie and the lanes were excised and digested with trypsin as described elsewhere (Shevchenko et al., 1996). In brief, proteins were reduced in 10 mM DTT for 30 min at 37°C, alkylated in 55 mM

iodoacetamide for 20 min at room temperature in the dark, and digested overnight at 37°C with 12.5 ng/µl Trypsin (Proteomics Grade, Sigma). The digestion media was then acidified to 0.1% of TFA and spun onto StageTips as described (Rappsilber et al., 2003). Peptides were eluted in 20 µl of 80% acetonitrile in 0.1% TFA and were concentrated to 4 µl (Concentrator 5301, Eppendorf AG). The peptides sample was then diluted to 5 µl by 0.1% TFA for LC-MS/MS analysis. Analyses were performed in a Velos LTQ-Orbitrap mass spectrometer (Thermofisher Scientific) coupled on-line to a Waters Nano AQUITY UPLC (Waters). Injections were performed in an analytical column with a self-assembled particle frit (Ishihama et al. 2002) and C18 material (ReproSil-Pur C18-AQ 3 µm; Dr. Maisch, GmbH) was packed into a spray emitter (75-µm ID, 8-µm opening, 300-mm length; New Objective) using an air-pressure pump (Proxeon Biosystems). Mobile phase A consisted of water, and 0.1% formic acid; mobile phase B, consisted of acetonitrile and 0.1% formic acid. The gradient used was 90 min. The peptides were loaded onto the column at a flow rate of 0.6 µl/min and eluted at a flow rate of 0.3 µl/min according to the gradient: 1 to 5% buffer B for 1 min, then to 32% B for 79 min, then to 76% B for 11 min and to 85% B for 1 min. FTMS spectra were recorded at 60,000 resolution and the twenty most intense peaks of the MS scan were selected in the ion trap for MS2, (normal scan, wideband activation, filling 5.0E5 ions for MS scan, 1.0E4 ions for MS2, maximum fill time 100 msec, dynamic exclusion for 60s sec). Searches were conducted against a database containing Mouse sequences (SwissProt) and the search parameters were: MS accuracy, 6 ppm; MS/MS accuracy, 0.6 Da; enzyme, trypsin; allowed number of missed cleavages, 2; fixed modification, carbamidometylation on Cysteine; variable modification, oxidation on Methionine.

Antibodies

Antibodies used for Western blots: anti-G9a mouse monoclonal (R&D Europe); anti-GLP mouse monoclonal (R&D Europe); anti-DNMT3A mouse monoclonal ab13888 (Abcam); anti-DNMT3B rabbit polyclonal PA1-884 (Thermo Scientific); anti-DNMT3B mouse monoclonal ab13604 (Abcam); anti-HDAC1 rabbit polyclonal sc-7872 (Santa Cruz Biotechnology); anti-Ran monoclonal (BD Biosciences); anti-H3K9me3 rabbit polyclonal 07-523 (Millipore); anti-H3K9me2 mouse monoclonal ab1220 (Abcam); anti-H3K9me1 07-450 (Millipore); anti-H4 rabbit polyclonal 07-108 (Millipore).

List of primers

Bisulfite DNA sequencing

Igf2r DMR2	Forward:	TTAGTGGGGTATTTTTATTTGTATGG
	Forward nested:	GTGTGGTATTTTTATGTATAGTTAGG
	Reverse:	AAATATCCTAAAAATACAAACTACAC

Igf2/H19 ICR	Forward:	GGTTTTTTGGTTATTGAATTTTAAAAATTA
	Forward nested:	TTAGTG TGGTTTATTATAGGAAGGTATAGAAGT
	Reverse:	TAAACCTAAAATACTCAAACTTTATCACAAC
	Reverse nested:	AAAAACCATTCCCTAAAATATCACAAATAC
Snrpn ICR	Forward:	GGGTTGTTAAAAATTTTAATAAGTTTAAAT
	Forward nested:	TTTAGAATGTTTTGGTTAAATAGGATGTAT
	Reverse:	AAAAAAACAAAAACCCCTACATTAC
Mest ICR	Forward:	GATTTGGGATATAAAAGGTTAATGAG
	Forward nested:	TTTTAGATTTTGAGGGTTTTAGGTTG
	Reverse:	TCATTAAAAACACAAACCTCCTTTAC
	Reverse nested:	AATCCCTTAAAAATCATCTTTCACAC
Kv ICR	Forward:	TTAGGTTTATAGAAGTAGGGGTGGT
	Reverse:	CTACAAAACTCAAAAATCTCCAAAC
Ankrd 50	Forward:	GGATGTGGTGGATTTGTTGTTA
	Forward nested:	TTGTTGTTAGAAGGAGGAGTAGATGT
	Reverse:	TCCAAACCTCTATCCAAAAAAATAC

Chromatin and methylated DNA immunoprecipitation (ChIP and MeDIP)

Dpep3	Forward:	GAAGTAACACCCCCAGCAGGGACA
	Reverse:	CCCTGAATCGAAGGTCCGAACCCA
Ankrd 50	Forward:	TCTCGTCCAAGCCTCTGTC
	Reverse:	GTCGATCACACCGATAACAAC
Igf2r ICR	Forward:	CGTGATCCTTGGTTGTGCTGAG
	Reverse:	CCAACCGGAATCGCATTAAAACC
Igf2/H19 ICR	Forward:	GGTGGCAGCATACTCCTATAT
	Reverse:	CTCGGCAACTTCGGTCTTAC
Snrpn ICR	Forward:	CAGGACATTCCGGTCAGAG
	Reverse:	TACTAGAATCCACAAGCCCAG
Zac1 ICR	Forward:	GCATCTGCGATTTGTCACTC
	Reverse:	CTTGCTCTCCAGTCCCGATA
Peg3 ICR	Forward:	CAGAGGACCCTGACAAGGAG
	Reverse:	AGCACAGCACTCTACGCACA
Peg10 ICR	Forward:	TCCTGACCAACTACGACCTG
	Reverse:	CCATACTCACCACACGAGGA
Peg13 ICR	Forward:	AGCTGAGCGAACCCCTTTAC
	Reverse:	CGCAGGTCTTCTATCCAACC
Kcnq1ot1 ICR	Forward:	CAGCACGGATCACTCCAG
	Reverse:	AAAGCTCTCCAAGTAGAATCACA

Rasgrf1 ICR	Forward:	CTGCACTTCGCTACCGTTTC
	Reverse:	AGTAGCAGTCGTGGTAGTTG
GnasXL ICR	Forward:	CACTGAGACCTGCGTCCTCT
	Reverse:	TGGTCGGCCAACAACTTTAG
Dlk ICR	Forward:	AGCGGCAGTGAGAATGAGAT
(IG-DMR)	Reverse:	TAAAGGCAAGACGAATCACAAGA
Mest	Forward:	TTTGGGGTGTTTTATGTCTTCCAG
	Reverse:	TCTCTAATCCTGAACCCCAGATTC
Mcts2	Forward:	TGAAGAAGAACCAGTGGGGTAATC
	Reverse:	CGGTCTACAGGAAGGATGGCAC
Impact	Forward:	TAATCCAAACTCTCCCATGGCTTC
	Reverse:	TTTCTGAAAGTGTTGGGAGTGACG
Ndufa	Forward:	TCCGCACCGTTACTCGCACG
	Reverse:	AGCCACCGTCGCTTCCTCCT
Wfdc15	Forward:	CCTGTTTTTCAAGGCTAAAGAGGG
	Reverse:	CCACCTTTGTTCTCTCGTTTTCTC

Quantitative RT PCR:

G9a	Forward:	ACACGGCATGGGATCTGACCCC
	Reverse:	ACGGCTCCCCATCCACACCATT
GAPDH	Forward:	GGCTCATGACCACAGTCCATGCC
	Reverse:	CACGGAAGGCCATGCCAGTGAG

wt G9a when shR forms are expressed

G9a	Forward:	GGTTTACTGCATTGATGCCCG
	Reverse:	TCGGTCACCGTAGTCAAAGC
	Forward:	TGGCGAGGTTTACTGCATTG
	Reverse:	CCCAGAATCGGTCACCGTAG

References

Ramsahoye, B.H. (2002). Measurement of genome wide DNA methylation by reversed-phase high-performance liquid chromatography. Methods *27*, 156-161.

Rappsilber, J., Ishihama, Y., and Mann, M. (2003). Stop and go extraction tips for matrix-assisted laser desorption/ionization, nanoelectrospray, and LC/MS sample pretreatment in proteomics. Anal Chem *75*, 663-670.

Shevchenko, A., Wilm, M., Vorm, O., and Mann, M. (1996). Mass spectrometric sequencing of proteins silver-stained polyacrylamide gels. Anal Chem *68*, 850-858.
